# Establishing a national laboratory quality system for HIV diagnosis and monitoring in resource-limited settings: Experience from Senegal

**DOI:** 10.4102/ajlm.v5i2.440

**Published:** 2016-10-17

**Authors:** Mouhamed A.S. Mbengue, Moussa Sarr, Papa A. Diaw, Papa A.N. Diall, Maimouna D. Toure, Ndeye F.F.N. Faye, Bousso Gueye, Ndeye C.T. Kane, Souleymane Mboup

**Affiliations:** 1 Laboratory of Bacteriology and Virology, Cheikh Anta Diop University, CHU Aristide Le Dantec, Dakar, Senegal; 2 Westat, Rockville, Maryland, United States; 3 National Committee for the Control of AIDS, Dakar, Senegal; 4 Division for the Control of AIDS and STDs, Ministry of Health, Dakar, Senegal

## HIV situation in Senegal

Senegal is a Western African Country, with a population of around 14 million people and 14 administrative regions. Based on the 2013 census, the total population of the country is estimated at 14 799 859 inhabitants, with a median population age of 18 years old.^1^ Senegal, has a broad-based, pyramid-shaped age structure, which is characteristic of a population with a very high proportion of children and young people (Table 1). In 2013, the Crude Birth Rate was 37.2 per thousand, with a sex ratio at birth of around 105 men per 100 women. Additionally, approximately 43% of the population is under 15 years of age, with an infant mortality rate of 47 deaths per 1000 births, and an overall risk of dying between birth and five years estimated at 72 deaths per 1000. The life expectancy at birth was 64 years in 2013.^1^

**TABLE 1:** Key HIV and health statistics in Senegal.

Indicators	Statistics
HIV prevalence in population aged 15–49 years	0.7%
Total number of people living with HIV	44 000 [37 000–53 000]
Estimated rate of mother-to-child HIV transmission	4.3%
Total number of babies born to HIV-positive mothers and on antiretroviral prophylaxis	638
Total number of pregnant women on antiretroviral prophylaxis	1371
Total number of pregnant women who had a CD4 count during pregnancy	914
Life expectancy at birth (years)	64
Infant mortality rate (per 1000 live births)	47
Maternal mortality ratio (per 100 000 live births)	320
Under-five mortality rate (per 1000 live births)	55
Death due to HIV (per 100 000 population)	13.7

**FIGURE 1:**
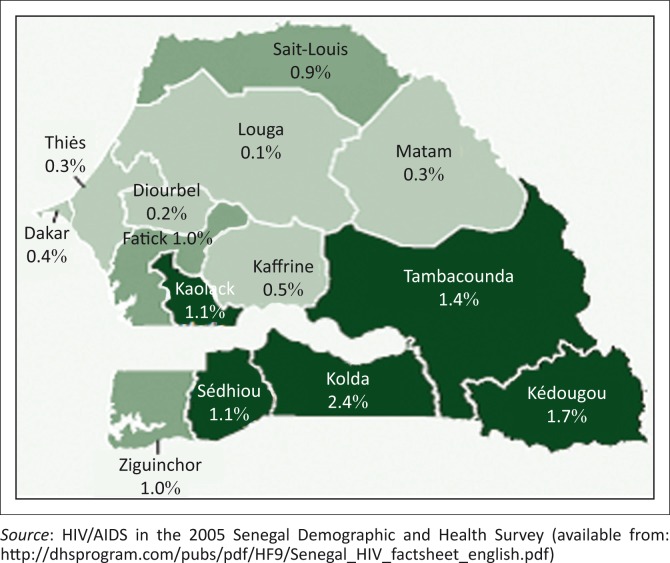
HIV prevalence rate per region in Senegal.

Senegal has a concentrated HIV epidemic with high prevalence rates in most-at-risk groups, including commercial sex workers, but low prevalence rates in the general population in most regions (Figure 1). Based on the 2010–2011 Senegal Demographic and Health Survey, the global prevalence of HIV amongst women and men aged 15–49 years is 0.7%.^2^ More women are infected with HIV than men; the sex ratio is typically around 60 men per 100 women infected. However, the HIV seroprevalence is higher among vulnerable populations, with rates at 18.5% among sex workers, 17.8% among men having sex with men, and 9.4% among injection drug users.^3,4^ Senegal has adopted the World Health Organization–Joint United Nations Programme on HIV/AIDS recommended 90-90-90 targets.^5^ The adoption of this strategy means that the country is expected, by 2020, to have 90% of its population living with HIV diagnosed, 90% of all those diagnosed receiving sustained HIV treatment, and 90% of those receiving antiretroviral therapy having suppressed viral load measures.^5^ To achieve these outcomes, having good clinical laboratory services for diagnosis and follow-up will be critical.^6^ More specifically, investments will be needed to improve laboratory infrastructure, and to facilitate the access and availability of routine viral load and early infant diagnosis (EID) measures through the implementation of point-of-care (POC) diagnostic platforms along with an efficient and sustainable quality assurance programme.

## Laboratory infrastructure in Senegal

Diagnostic laboratories in Senegal operate within a three-tiered laboratory system: (1) district and peripheral health centre level laboratories; (2) regional level laboratories; and (3) central and hospital level laboratories (Table 2). The organisation of the laboratory system is part of the overall health system structure and is placed under the leadership of the Ministry of Health. There is a Bureau of Laboratories within the Ministry of Health that is responsible for the implementation of policies defined by the government concerning the functioning and organisation of clinical laboratories. This office also promotes good clinical laboratory practice, not only for public medical laboratories, but also for private laboratories within the country.

**TABLE 2:** Existing technologies or platforms HIV diagnosis, CD4 count, and viral load monitoring at each level of the tiered health system in Senegal.

Laboratory	Level	Activities	Existing technologies or platforms		
			CD4 testing	Viral load testing	HIV testing
LBV/CADU	-	HIV rapid diagnostic test and confirmation CD4 count. Viral load monitoring and early infant diagnosis. Validating diagnostic testing. Validating POC testing. Coordination, supervision and quality assurance. Proficiency testing.	BD FacsCount^®^. Apogee Auto40^®^. BD FacsCalibur ^®^. Pima CD4^®^. Coulter^®^ Cyflow counter^®^ Point care Now^®^. Millipore Guava^®^. Cyflow ^®^ Minipoc^®^	Cobas TaqMan^®^. Abbott *m*2000^®^. NucliSENS Easy Q V2.0^®^. NucliSENS Easy Mag^®^ NucliSENS MiniMag^®^	-
Regional or hospital laboratories	3	HIV rapid diagnostic testing and confirmation CD4 count Viral load and EID	-	Abbott *m*2000^®^. NucliSENS Easy Q V2.0^®^. Cobas TaqMan^®^.	POC and cytometers with an average of three per region
District-level laboratories	2	HIV rapid diagnostic test and confirmation. CD4 count. Collecting DBS for EID	POC and cytometers at an average of three per region	-	Rapid screening test
Primary healthcare worker	1	HIV rapid diagnostic test Collecting DBS for EID	-	-	Rapid screening test

EID, early infant diagnosis; DBS, Dried blood spot; POC, Point of care; LBV/CADU, Laboratory of bacteriology and Virology/Cheikh Anta Diop University.

The Laboratory of Bacteriology and Virology (LBV) at Cheikh Anta Diop University (CADU), which is located at Le Dantec Hospital in the capital city, has been recognised by health authorities as the HIV National Reference laboratory since 1986. In partnership with the Ministry of Health and the National AIDS programme, LBV/CADU is responsible for evaluating and validating diagnostic testing technologies, and technologies for CD4 count, viral load and EID. Additionally, LBV/CADU ensures the supervision of other laboratories through a national external quality assessment (EQA) programme, through distribution of regular proficiency testing panels, collection of data for analysis, and follow-up and corrective actions in partnership with the Ministry of Health, the National Committee for the Control of AIDS and the Division for the Control of AIDS and Sexually Transmitted Diseases.

At the central and reference laboratory level, the HIV viral load, EID, CD4-count monitoring and HIV-diagnosis capabilities of LBV/CADU are described in Table 2. At the regional and hospital laboratories level, these infrastructures are capable of performing HIV rapid diagnostic test confirmation, CD4-count measures and, at some of them, viral load and EID testing. However, the vast majority of the laboratory activities are conducted at the district level and peripheral health centre level. The capabilities for the district/peripheral health centre-level laboratories include: HIV rapid testing, CD4 count and collection of dried-blood specimens for EID. There are also few private laboratories operating at different levels of the health system.

## Quality assurance framework

Quality assurance is the backbone of quality laboratory performance^7^ and proficiency testing is one of the major components of a quality assurance programme. In Senegal, laboratory infrastructure is relatively well developed at the national and regional levels. However, issues such as inadequate infrastructure, shortage of qualified staff, lack of equipment, limited quality assurance and control procedures are frequently seen among district and peripheral health centre laboratories.

The maintenance of a quality management system, including quality assurance, is crucial for having and providing good and reliable laboratory services.^7,8,9^ LBV/CADU has been working recently with the US Centers for Disease Control and Prevention to strengthen laboratory systems in Africa and Senegal though the implementation of quality management systems. The LBV/CADU-hosted AFRIQUALAB has been created for that purpose. AFRIQUALAB aims to improve laboratory quality in Africa through regular organisation and distribution of proficiency testing panels across the African continent, including in francophone countries. AFRIQUALAB works in partnership with the US Centers for Disease Control and Prevention, Westat and One World Accuracy, a private Canadian-based organisation specialising in EQA. During 2015, there were 174 participating laboratories from 28 countries, of which eight laboratories were from Senegal. The proficiency testing organized by AFRIQUALAB is mostly free and focused on HIV-related proficiency testing panels, including HIV serology and flow cytometry. Through technology transfer from the US Centers for Disease Control and Prevention, the programme is also offering free EQA for EID using dried-blood specimens and for HIV viral load using dried-tube specimens. Currently, more than 60 laboratories are participating in this EQA sub-programme for EID using dried-blood specimens and for HIV viral load using dried-tube specimens in Africa, including eight laboratories in Senegal.

Additionally, AFRIQUALAB is also involved in an EQA programme focusing only on CD4 count technologies within Senegal, with the support of the National AIDS programme and the Public Health Agency of Canada, an international programme for quality assessment and standardisation for immunological measures relevant to HIV. AFRIQUALAB also offers other panels, such as haematology, biochemistry, hepatitis B and C and mycobacteria, to participating labs using a fee-based service structure. LBV/CADU has made major improvements to its own quality management system and has recently achieved International Organization for Standardization 15189 accreditation of its medical laboratories.

Additionally, within Senegal and at the national level, the Ministry of Health has recently released a national strategic plan that will guide the quality management system across the medical laboratory services and system in the country.

## Lessons learned and challenges

Laboratories participating in the EQA programme for HIV rapid testing and CD4 (including POC) received feedback and, based on their results, have implemented corrective actions, as needed, to improve the quality of services, and were encouraged to participate in a laboratory network for continuous improvement. These outcomes are positive steps toward the implementation of quality management systems.

The feedback received from the EQA programme has been used as an opportunity for participating laboratories to be more vigilant in many aspects of their work; for example, with respect to the expiration date for reagents, implementation of corrective actions, and on-site training for staff. The quality assurance programme and the external quality control activities must be followed up by systems-strengthening activities, such as staff training at all levels. We found that there is a need for more communication between the National Reference Laboratories and the Bureau of Laboratories, located at the Ministry of Health, regarding the type of corrective actions and support to be provided from the central/national level to regional and peripheral laboratories. There is also a need to provide incentives such as certificates of participation or achievement to successfully performing labs.

## Quality assurance for POC

Currently, all 24 sites in-country with POC diagnostic technologies, including POC for CD4, are participating in an EQA programme organised by LBV. Since 2004, LBV, with the support of the national AIDS programme, has provided voluntary, free-of-charge EQA of HIV rapid testing to assess the performance of laboratories conducting HIV diagnostics in Dakar and other regions. Currently, the EQA programme for HIV rapid testing uses dried-tube specimens. The proficiency testing panels consist of four specimens (two negatives, one HIV-1, one HIV-2) distributed at ambient temperature to participants. The results from the participants are sent to the national reference laboratory via email or cell-phone text messaging.

### Costing and cost-effectiveness of HIV POC testing

With regard to the 90-90-90 goals, the affordability of viral load testing and CD4 count measures is a key factor in plans to expand HIV laboratory services and scaling antiretroviral therapy across Senegal. The country will need to put in place an affordable and sustainable strategy to reinforce the capacity of the public laboratories as well as to advocate for a strong political commitment from the Ministry of Health and sponsors. In partnership with the National AIDS programme, LBV/CADU is planning to estimate the costing and to undertake a cost-effectiveness study of implementing a nationwide HIV POC testing system, for CD4 count and viral load testing. The costing for, and cost-effectiveness of, a related quality assurance programme will be also taken into account. A model of costing for HIV POC testing, including quality assurance activities is currently under review with the International Diagnostics Centre of the London School of Hygiene and Tropical Medicine.
